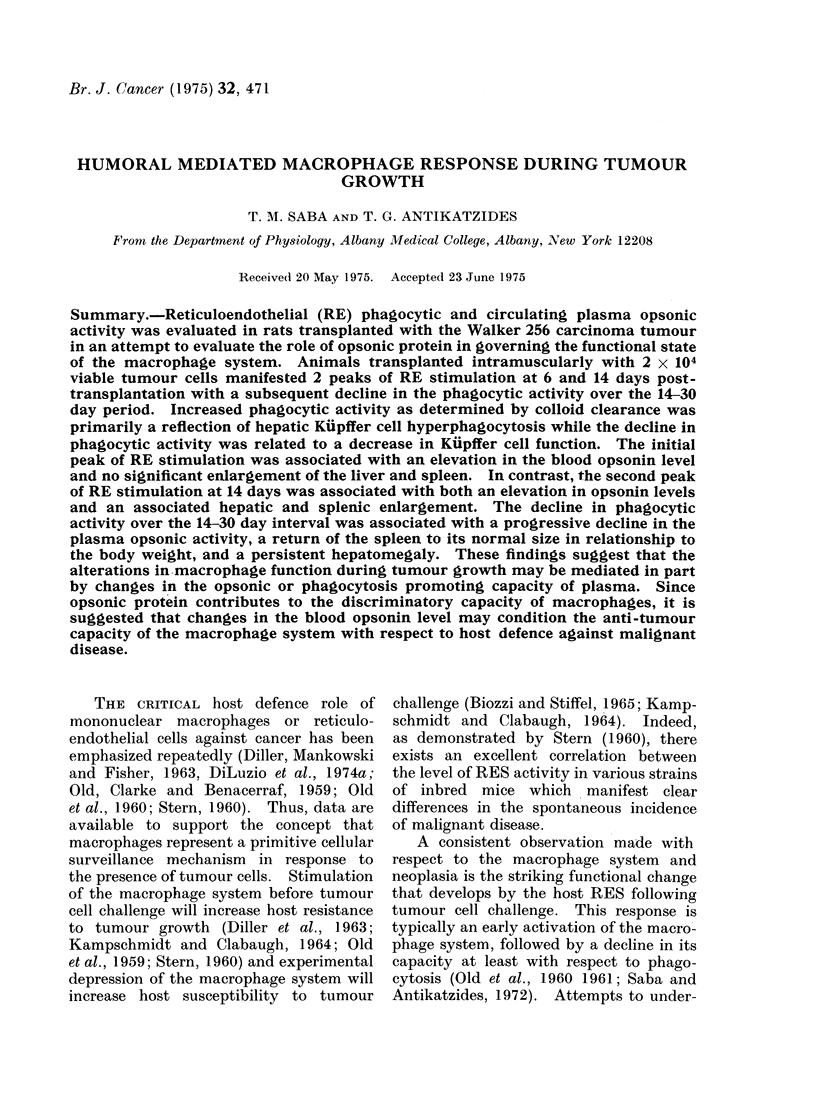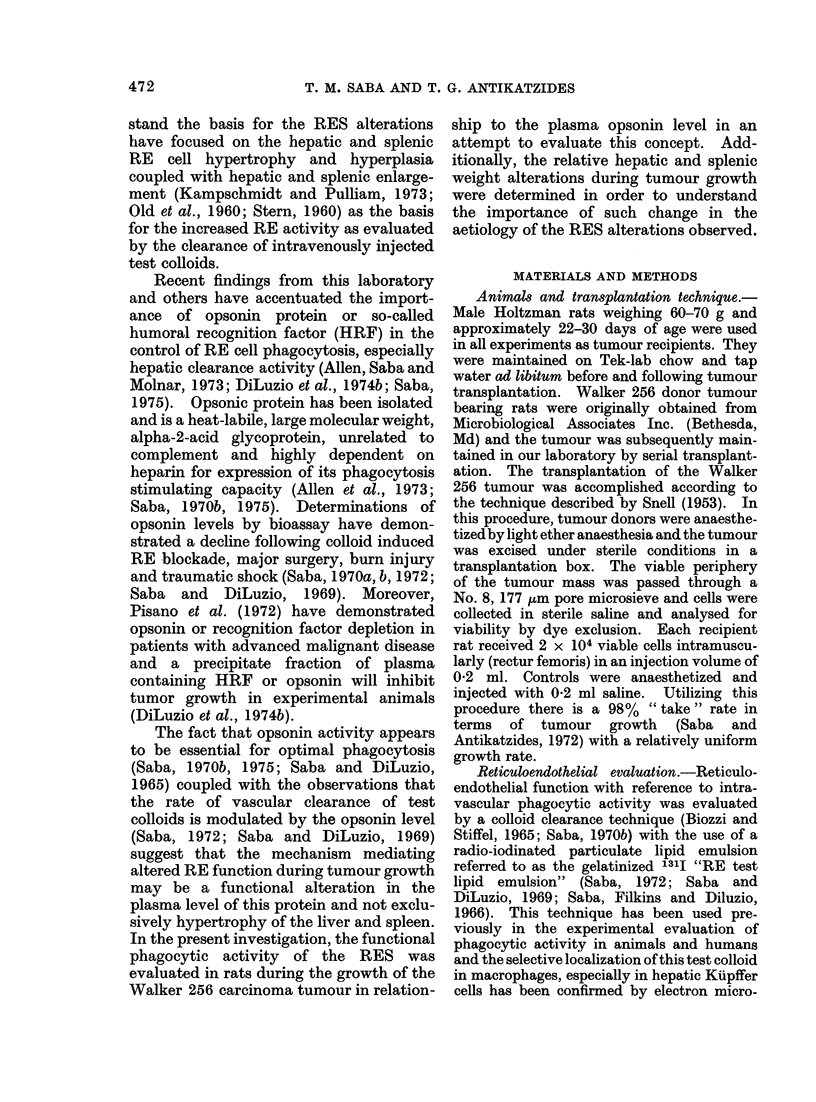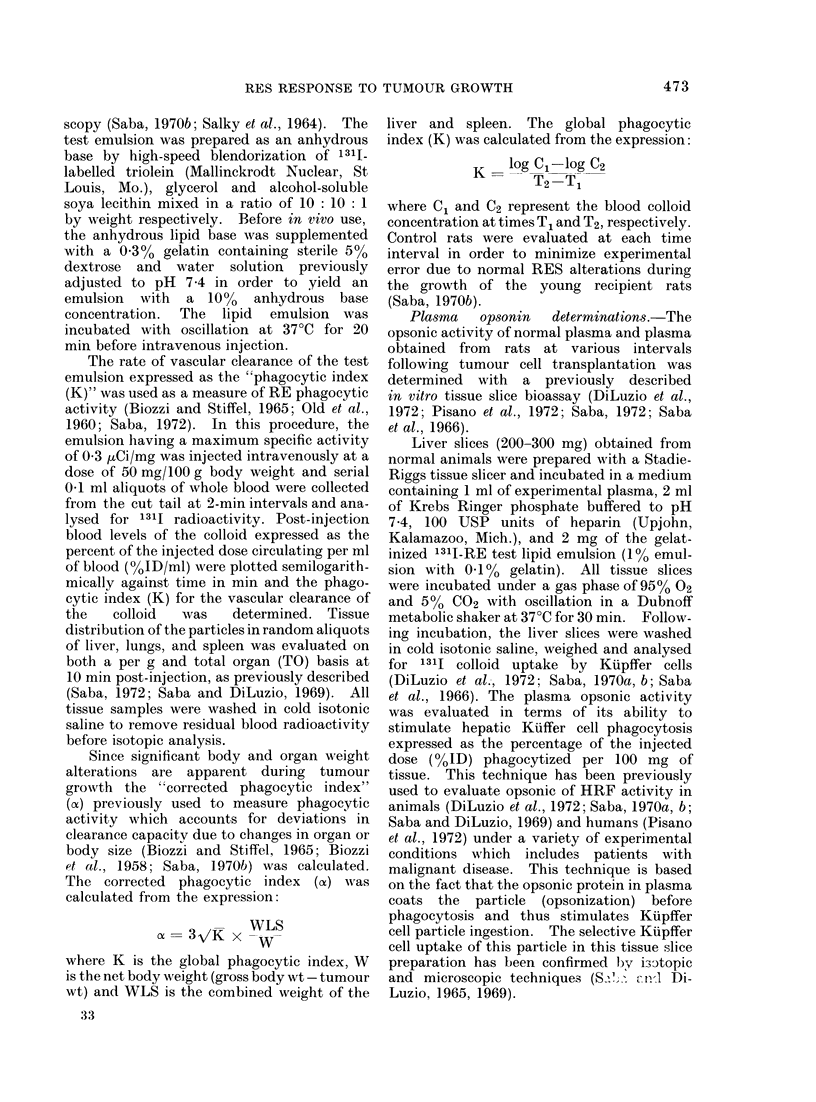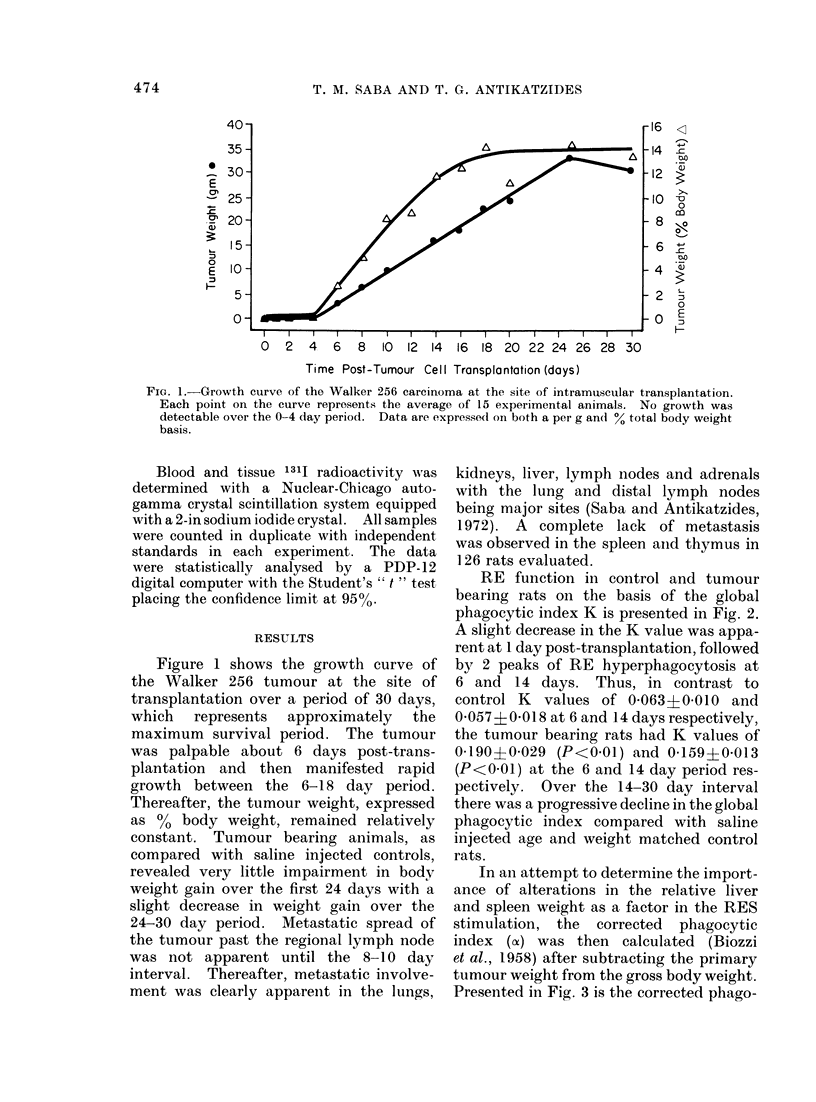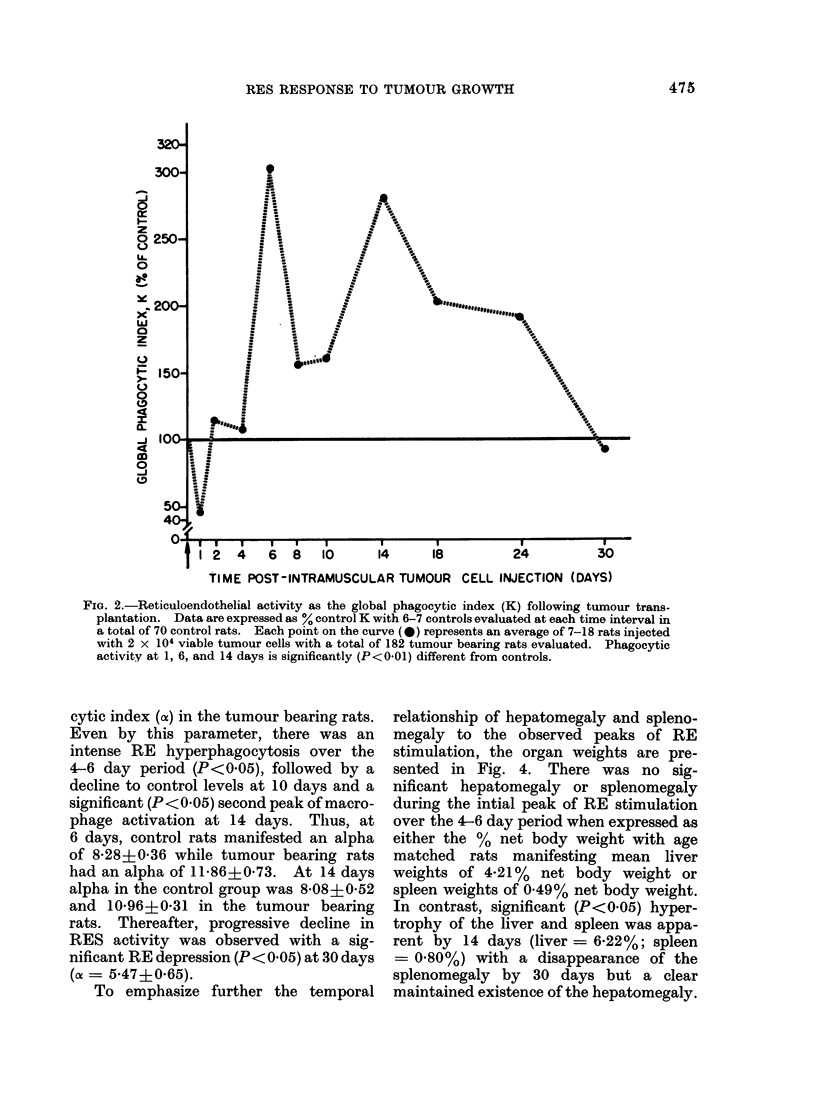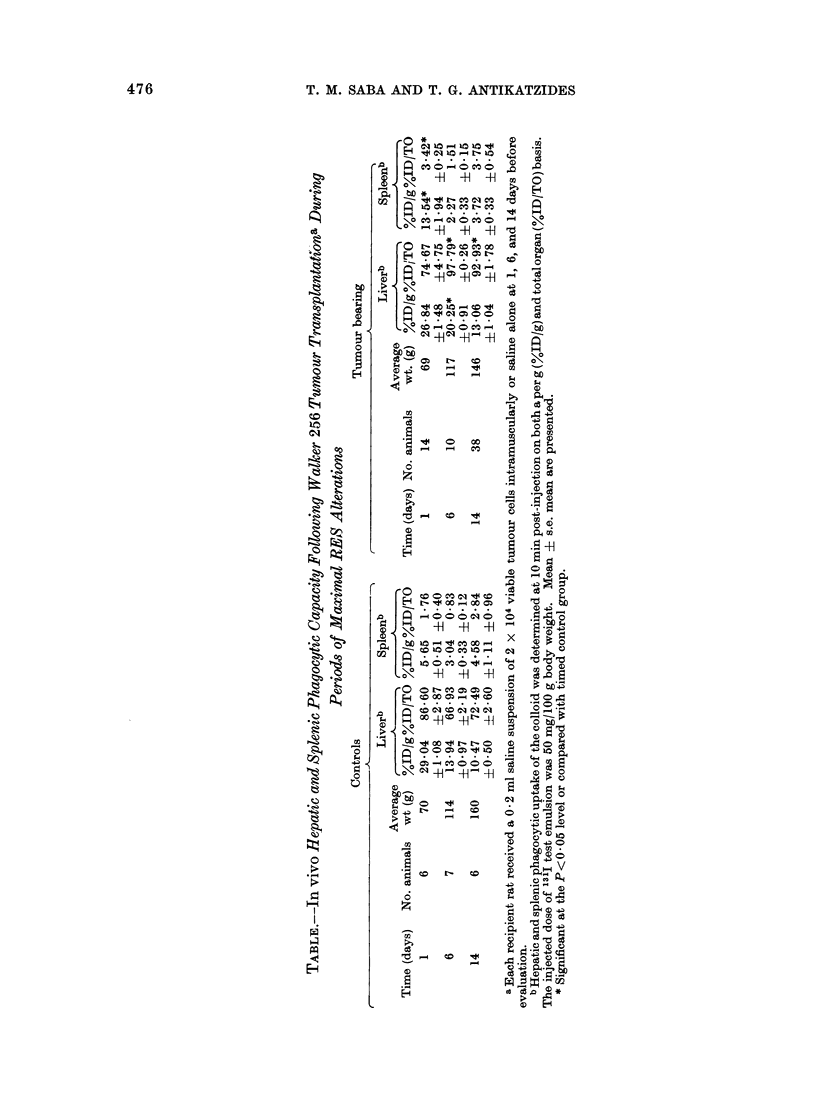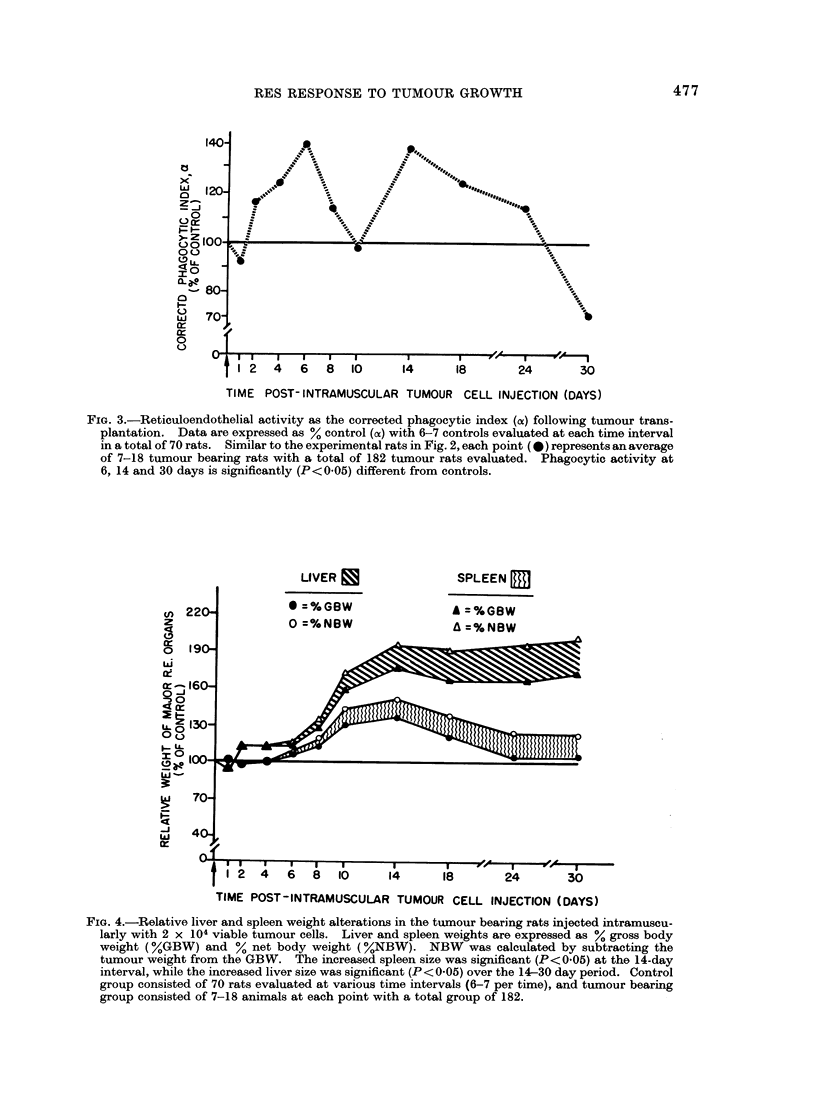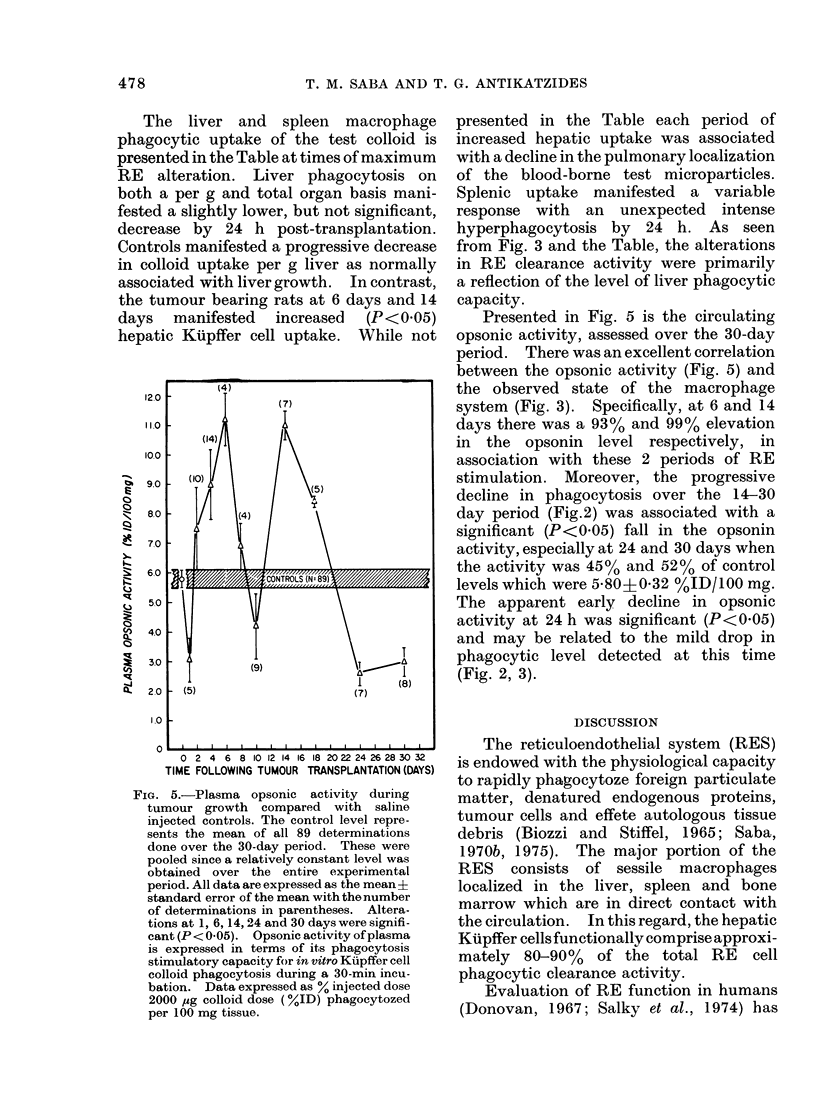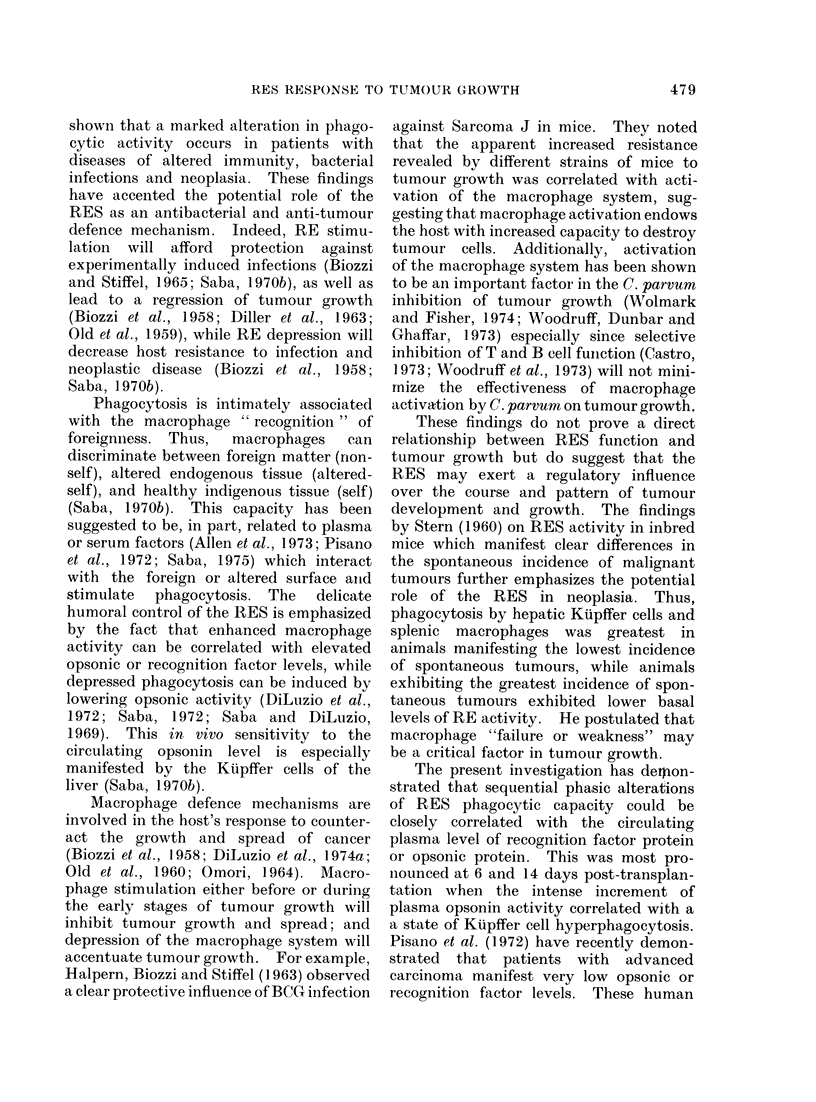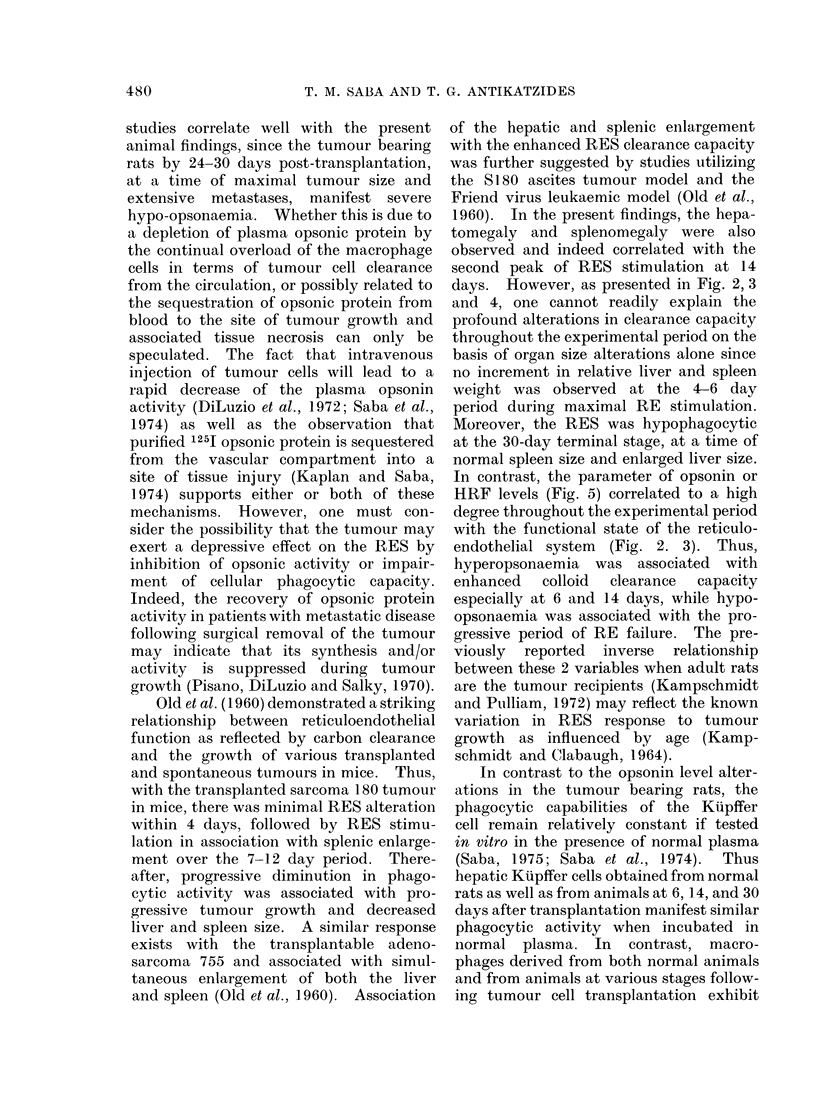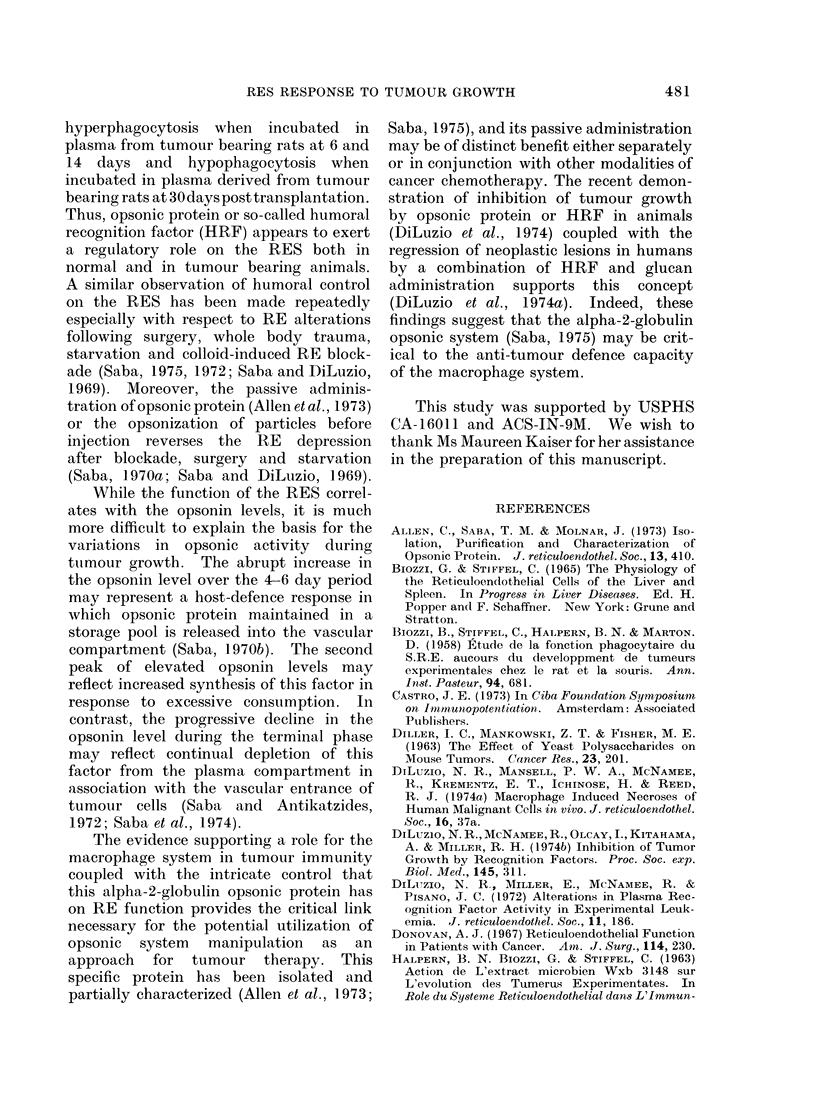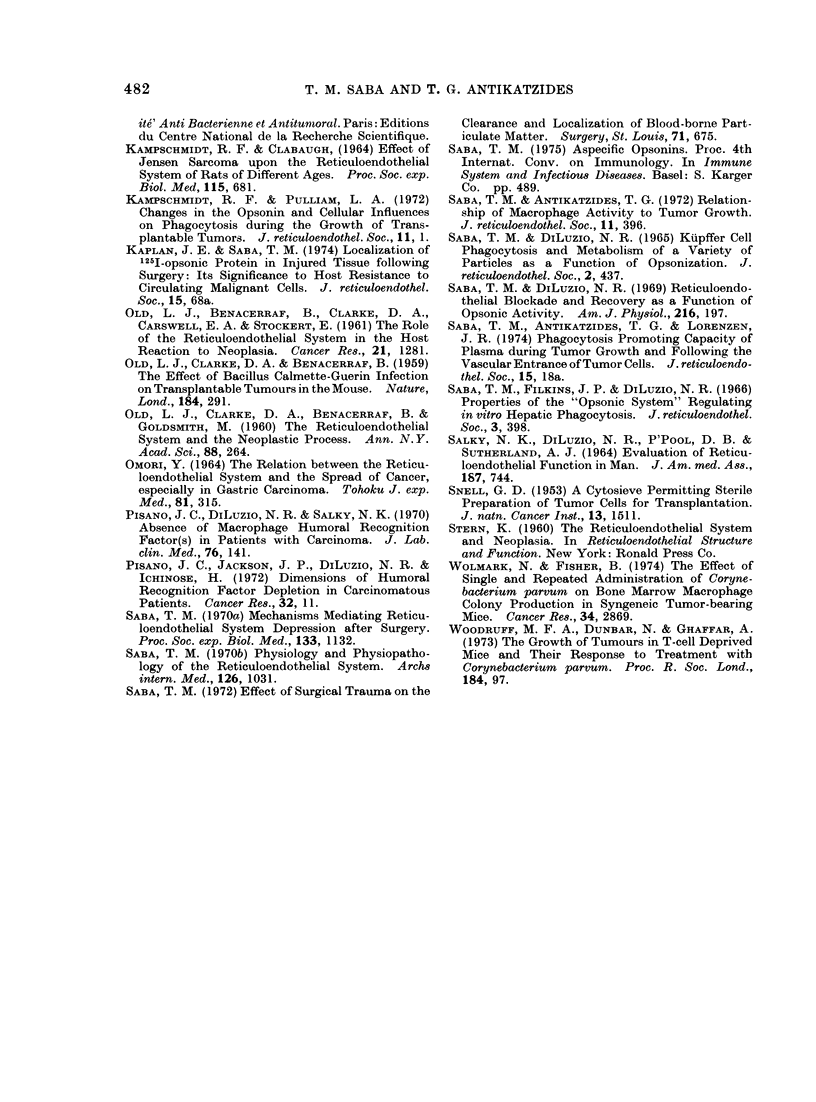# Humoral mediated macrophage response during tumour growth.

**DOI:** 10.1038/bjc.1975.249

**Published:** 1975-10

**Authors:** T. M. Saba, T. G. Antikatzides

## Abstract

Reticuloendothelial (RE) phagocytic and circulating plasma opsonic activity was evaluated in rats transplanted with the Walker 256 carcinoma tumour in an attempt to evaluate the role of opsonic protein in governing the functional state of the macrophage system. Animals transplanted intramuscularly with 2 X 10(4) viable tumour cells manifested 2 peaks of RE stimulation at 6 and 14 days post-transplantation with a subsequent decline in the phagocytic activity over the 14-30 day period. Increased phagocytic activity as determined by colloid clearance was primarily a reflection of hepatic Küpffer cell hyperphagocytosis while the decline in phagocytic activity was related to a decrease in Küpffer cell function. The initial peak of RE stimulation was associated with an elevation in the blood opsonin level and no significant enlargement of the liver and spleen. In contrast, the second peak of RE stimulation at 14 days was associated with both an elevation in opsonin levels and an associated hepatic and splenic enlargement. The decline in phagocytic activity over the 14-30 day interval was associated with a progressive decline in the plasma opsonic activity, a return of the spleen to its normal size in relationship to the body weight, and a persistent hepatomegaly. These findings suggest that the alterations in macrophage function during tumour growth may be mediated in part by changes in the opsonic or phagocytosis promoting capacity of plasma. Since opsonic protein contributes to the discriminatory capacity of macrophages, it is suggested that changes in the blood opsonin level may condition the anti-tumour capacity of the macrophage system with respect to host defence aginst malignant disease.


Br. J. Cancer (1975) **32**, 471

### HUMORAL MEDIATED MACROPHAGE RESPONSE DURING TUMOUR GROWTH

T. M. SABA AND T. G. ANTIKATZIDES

*From the Department of Physiology, Albany Medical College, Albany, New York* 12208

Received 20 May 1975. Accepted 23 June 1975

**Summary.—Reticuloendothelial (RE) phagocytic and circulating plasma opsonic activity was evaluated in rats transplanted with the Walker 256 carcinoma tumour in an attempt to evaluate the role of opsonic protein in governing the functional state of the macrophage system. Animals transplanted intramuscularly with 2 $\times$ 10$^4$ viable tumour cells manifested 2 peaks of RE stimulation at 6 and 14 days post-transplantation with a subsequent decline in the phagocytic activity over the 14–30 day period. Increased phagocytic activity as determined by colloid clearance was primarily a reflection of hepatic Küpffer cell hyperphagocytosis while the decline in phagocytic activity was related to a decrease in Küpffer cell function. The initial peak of RE stimulation was associated with an elevation in the blood opsonin level and no significant enlargement of the liver and spleen. In contrast, the second peak of RE stimulation at 14 days was associated with both an elevation in opsonin levels and an associated hepatic and splenic enlargement. The decline in phagocytic activity over the 14–30 day interval was associated with a progressive decline in the plasma opsonic activity, a return of the spleen to its normal size in relationship to the body weight, and a persistent hepatomegaly. These findings suggest that the alterations in macrophage function during tumour growth may be mediated in part by changes in the opsonic or phagocytosis promoting capacity of plasma. Since opsonic protein contributes to the discriminatory capacity of macrophages, it is suggested that changes in the blood opsonin level may condition the anti-tumour capacity of the macrophage system with respect to host defence against malignant disease.**

THE CRITICAL host defence role of mononuclear macrophages or reticulo-endothelial cells against cancer has been emphasized repeatedly (Diller, Mankowski and Fisher, 1963, DiLuzio *et al.*, 1974*a;* Old, Clarke and Benacerraf, 1959; Old *et al.*, 1960; Stern, 1960). Thus, data are available to support the concept that macrophages represent a primitive cellular surveillance mechanism in response to the presence of tumour cells. Stimulation of the macrophage system before tumour cell challenge will increase host resistance to tumour growth (Diller *et al.*, 1963; Kampschmidt and Clabaugh, 1964; Old *et al.*, 1959; Stern, 1960) and experimental depression of the macrophage system will increase host susceptibility to tumour challenge (Biozzi and Stiffel, 1965; Kampschmidt and Clabaugh, 1964). Indeed, as demonstrated by Stern (1960), there exists an excellent correlation between the level of RES activity in various strains of inbred mice which manifest clear differences in the spontaneous incidence of malignant disease.

A consistent observation made with respect to the macrophage system and neoplasia is the striking functional change that develops by the host RES following tumour cell challenge. This response is typically an early activation of the macrophage system, followed by a decline in its capacity at least with respect to phagocytosis (Old *et al.*, 1960 1961; Saba and Antikatzides, 1972). Attempts to understand the basis for the RES alterations have focused on the hepatic and splenic RE cell hypertrophy and hyperplasia coupled with hepatic and splenic enlargement (Kampschmidt and Pulliam, 1973; Old *et al.*, 1960; Stern, 1960) as the basis for the increased RE activity as evaluated by the clearance of intravenously injected test colloids.

Recent findings from this laboratory and others have accentuated the importance of opsonin protein or so-called humoral recognition factor (HRF) in the control of RE cell phagocytosis, especially hepatic clearance activity (Allen, Saba and Molnar, 1973; DiLuzio *et al.*, 1974*b*; Saba, 1975). Opsonic protein has been isolated and is a heat-labile, large molecular weight, alpha-2-acid glycoprotein, unrelated to complement and highly dependent on heparin for expression of its phagocytosis stimulating capacity (Allen *et al.*, 1973; Saba, 1970*b*, 1975). Determinations of opsonin levels by bioassay have demonstrated a decline following colloid induced RE blockade, major surgery, burn injury and traumatic shock (Saba, 1970*a*, *b*, 1972; Saba and DiLuzio, 1969). Moreover, Pisano *et al.* (1972) have demonstrated opsonin or recognition factor depletion in patients with advanced malignant disease and a precipitate fraction of plasma containing HRF or opsonin will inhibit tumor growth in experimental animals (DiLuzio *et al.*, 1974*b*).

The fact that opsonin activity appears to be essential for optimal phagocytosis (Saba, 1970*b*, 1975; Saba and DiLuzio, 1965) coupled with the observations that the rate of vascular clearance of test colloids is modulated by the opsonin level (Saba, 1972; Saba and DiLuzio, 1969) suggest that the mechanism mediating altered RE function during tumour growth may be a functional alteration in the plasma level of this protein and not exclusively hypertrophy of the liver and spleen. In the present investigation, the functional phagocytic activity of the RES was evaluated in rats during the growth of the Walker 256 carcinoma tumour in relationship to the plasma opsonin level in an attempt to evaluate this concept. Additionally, the relative hepatic and splenic weight alterations during tumour growth were determined in order to understand the importance of such change in the aetiology of the RES alterations observed.

### MATERIALS AND METHODS

*Animals and transplantation technique.*—Male Holtzman rats weighing 60–70 g and approximately 22–30 days of age were used in all experiments as tumour recipients. They were maintained on Tek-lab chow and tap water *ad libitum* before and following tumour transplantation. Walker 256 donor tumour bearing rats were originally obtained from Microbiological Associates Inc. (Bethesda, Md) and the tumour was subsequently maintained in our laboratory by serial transplantation. The transplantation of the Walker 256 tumour was accomplished according to the technique described by Snell (1953). In this procedure, tumour donors were anaesthetized by light ether anaesthesia and the tumour was excised under sterile conditions in a transplantation box. The viable periphery of the tumour mass was passed through a No. 8, 177 μm pore microsieve and cells were collected in sterile saline and analysed for viability by dye exclusion. Each recipient rat received $2 \times 10^4$ viable cells intramuscularly (rectur femoris) in an injection volume of 0·2 ml. Controls were anaesthetized and injected with 0·2 ml saline. Utilizing this procedure there is a 98% " take " rate in terms of tumour growth (Saba and Antikatzides, 1972) with a relatively uniform growth rate.

*Reticuloendothelial evaluation.*—Reticuloendothelial function with reference to intravascular phagocytic activity was evaluated by a colloid clearance technique (Biozzi and Stiffel, 1965; Saba, 1970*b*) with the use of a radio-iodinated particulate lipid emulsion referred to as the gelatinized $^{131}$I "RE test lipid emulsion" (Saba, 1972; Saba and DiLuzio, 1969; Saba, Filkins and Diluzio, 1966). This technique has been used previously in the experimental evaluation of phagocytic activity in animals and humans and the selective localization of this test colloid in macrophages, especially in hepatic Küpffer cells has been confirmed by electron microscopy (Saba, 1970*b*; Salky *et al.*, 1964). The test emulsion was prepared as an anhydrous base by high-speed blendorization of $^{131}$I-labelled triolein (Mallinckrodt Nuclear, St Louis, Mo.), glycerol and alcohol-soluble soya lecithin mixed in a ratio of 10 : 10 : 1 by weight respectively. Before *in vivo* use, the anhydrous lipid base was supplemented with a 0·3% gelatin containing sterile 5% dextrose and water solution previously adjusted to pH 7·4 in order to yield an emulsion with a 10% anhydrous base concentration. The lipid emulsion was incubated with oscillation at 37°C for 20 min before intravenous injection.

The rate of vascular clearance of the test emulsion expressed as the "phagocytic index (K)" was used as a measure of RE phagocytic activity (Biozzi and Stiffel, 1965; Old *et al.*, 1960; Saba, 1972). In this procedure, the emulsion having a maximum specific activity of 0·3 $\mu$Ci/mg was injected intravenously at a dose of 50 mg/100 g body weight and serial 0·1 ml aliquots of whole blood were collected from the cut tail at 2-min intervals and analysed for $^{131}$I radioactivity. Post-injection blood levels of the colloid expressed as the percent of the injected dose circulating per ml of blood (%ID/ml) were plotted semilogarithmically against time in min and the phagocytic index (K) for the vascular clearance of the colloid was determined. Tissue distribution of the particles in random aliquots of liver, lungs, and spleen was evaluated on both a per g and total organ (TO) basis at 10 min post-injection, as previously described (Saba, 1972; Saba and DiLuzio, 1969). All tissue samples were washed in cold isotonic saline to remove residual blood radioactivity before isotopic analysis.

Since significant body and organ weight alterations are apparent during tumour growth the "corrected phagocytic index" ($\alpha$) previously used to measure phagocytic activity which accounts for deviations in clearance capacity due to changes in organ or body size (Biozzi and Stiffel, 1965; Biozzi *et al.*, 1958; Saba, 1970*b*) was calculated. The corrected phagocytic index ($\alpha$) was calculated from the expression:

$$\alpha = 3\sqrt{K} \times \frac{WLS}{W}$$

where K is the global phagocytic index, W is the net body weight (gross body wt − tumour wt) and WLS is the combined weight of the liver and spleen. The global phagocytic index (K) was calculated from the expression:

$$K = \frac{\log C_1 - \log C_2}{T_2 - T_1}$$

where $C_1$ and $C_2$ represent the blood colloid concentration at times $T_1$ and $T_2$, respectively. Control rats were evaluated at each time interval in order to minimize experimental error due to normal RES alterations during the growth of the young recipient rats (Saba, 1970*b*).

*Plasma opsonin determinations.*—The opsonic activity of normal plasma and plasma obtained from rats at various intervals following tumour cell transplantation was determined with a previously described *in vitro* tissue slice bioassay (DiLuzio *et al.*, 1972; Pisano *et al.*, 1972; Saba, 1972; Saba *et al.*, 1966).

Liver slices (200–300 mg) obtained from normal animals were prepared with a Stadie-Riggs tissue slicer and incubated in a medium containing 1 ml of experimental plasma, 2 ml of Krebs Ringer phosphate buffered to pH 7·4, 100 USP units of heparin (Upjohn, Kalamazoo, Mich.), and 2 mg of the gelatinized $^{131}$I-RE test lipid emulsion (1% emulsion with 0·1% gelatin). All tissue slices were incubated under a gas phase of 95% $O_2$ and 5% $CO_2$ with oscillation in a Dubnoff metabolic shaker at 37°C for 30 min. Following incubation, the liver slices were washed in cold isotonic saline, weighed and analysed for $^{131}$I colloid uptake by Küpffer cells (DiLuzio *et al.*, 1972; Saba, 1970*a*, *b*; Saba *et al.*, 1966). The plasma opsonic activity was evaluated in terms of its ability to stimulate hepatic Küffer cell phagocytosis expressed as the percentage of the injected dose (%ID) phagocytized per 100 mg of tissue. This technique has been previously used to evaluate opsonic of HRF activity in animals (DiLuzio *et al.*, 1972; Saba, 1970*a*, *b*; Saba and DiLuzio, 1969) and humans (Pisano *et al.*, 1972) under a variety of experimental conditions which includes patients with malignant disease. This technique is based on the fact that the opsonic protein in plasma coats the particle (opsonization) before phagocytosis and thus stimulates Küpffer cell particle ingestion. The selective Küpffer cell uptake of this particle in this tissue slice preparation has been confirmed by isotopic and microscopic techniques (Saba and DiLuzio, 1965, 1969).

FIG. 1.—Growth curve of the Walker 256 carcinoma at the site of intramuscular transplantation. Each point on the curve represents the average of 15 experimental animals. No growth was detectable over the 0–4 day period. Data are expressed on both a per g and % total body weight basis.

Blood and tissue $^{131}I$ radioactivity was determined with a Nuclear-Chicago auto-gamma crystal scintillation system equipped with a 2-in sodium iodide crystal. All samples were counted in duplicate with independent standards in each experiment. The data were statistically analysed by a PDP-12 digital computer with the Student's " $t$ " test placing the confidence limit at 95%.

RESULTS

Figure 1 shows the growth curve of the Walker 256 tumour at the site of transplantation over a period of 30 days, which represents approximately the maximum survival period. The tumour was palpable about 6 days post-transplantation and then manifested rapid growth between the 6–18 day period. Thereafter, the tumour weight, expressed as % body weight, remained relatively constant. Tumour bearing animals, as compared with saline injected controls, revealed very little impairment in body weight gain over the first 24 days with a slight decrease in weight gain over the 24–30 day period. Metastatic spread of the tumour past the regional lymph node was not apparent until the 8–10 day interval. Thereafter, metastatic involvement was clearly apparent in the lungs, kidneys, liver, lymph nodes and adrenals with the lung and distal lymph nodes being major sites (Saba and Antikatzides, 1972). A complete lack of metastasis was observed in the spleen and thymus in 126 rats evaluated.

RE function in control and tumour bearing rats on the basis of the global phagocytic index K is presented in Fig. 2. A slight decrease in the K value was apparent at 1 day post-transplantation, followed by 2 peaks of RE hyperphagocytosis at 6 and 14 days. Thus, in contrast to control K values of $0{\cdot}063 \pm 0{\cdot}010$ and $0{\cdot}057 \pm 0{\cdot}018$ at 6 and 14 days respectively, the tumour bearing rats had K values of $0{\cdot}190 \pm 0{\cdot}029$ ($P < 0{\cdot}01$) and $0{\cdot}159 \pm 0{\cdot}013$ ($P < 0{\cdot}01$) at the 6 and 14 day period respectively. Over the 14–30 day interval there was a progressive decline in the global phagocytic index compared with saline injected age and weight matched control rats.

In an attempt to determine the importance of alterations in the relative liver and spleen weight as a factor in the RES stimulation, the corrected phagocytic index (α) was then calculated (Biozzi *et al.*, 1958) after subtracting the primary tumour weight from the gross body weight. Presented in Fig. 3 is the corrected phago-

FIG. 2.—Reticuloendothelial activity as the global phagocytic index (K) following tumour transplantation. Data are expressed as % control K with 6–7 controls evaluated at each time interval in a total of 70 control rats. Each point on the curve (●) represents an average of 7–18 rats injected with $2 \times 10^4$ viable tumour cells with a total of 182 tumour bearing rats evaluated. Phagocytic activity at 1, 6, and 14 days is significantly ($P<0{\cdot}01$) different from controls.

cytic index (α) in the tumour bearing rats. Even by this parameter, there was an intense RE hyperphagocytosis over the 4–6 day period ($P<0{\cdot}05$), followed by a decline to control levels at 10 days and a significant ($P<0{\cdot}05$) second peak of macrophage activation at 14 days. Thus, at 6 days, control rats manifested an alpha of $8{\cdot}28 \pm 0{\cdot}36$ while tumour bearing rats had an alpha of $11{\cdot}86 \pm 0{\cdot}73$. At 14 days alpha in the control group was $8{\cdot}08 \pm 0{\cdot}52$ and $10{\cdot}96 \pm 0{\cdot}31$ in the tumour bearing rats. Thereafter, progressive decline in RES activity was observed with a significant RE depression ($P<0{\cdot}05$) at 30 days ($\alpha = 5{\cdot}47 \pm 0{\cdot}65$).

To emphasize further the temporal relationship of hepatomegaly and splenomegaly to the observed peaks of RE stimulation, the organ weights are presented in Fig. 4. There was no significant hepatomegaly or splenomegaly during the intial peak of RE stimulation over the 4–6 day period when expressed as either the % net body weight with age matched rats manifesting mean liver weights of 4·21% net body weight or spleen weights of 0·49% net body weight. In contrast, significant ($P<0{\cdot}05$) hypertrophy of the liver and spleen was apparent by 14 days (liver = 6·22%; spleen = 0·80%) with a disappearance of the splenomegaly by 30 days but a clear maintained existence of the hepatomegaly.

TABLE.—In vivo *Hepatic and Splenic Phagocytic Capacity Following Walker 256 Tumour Transplantation*[a] *During Periods of Maximal RES Alterations*

| Controls | | | | | | | Tumour bearing | | | | | | |
|---|---|---|---|---|---|---|---|---|---|---|---|---|---|
| | | | Liver[b] | | Spleen[b] | | | | | Liver[b] | | Spleen[b] | |
| Time (days) | No. animals | Average wt (g) | %ID/g | %ID/TO | %ID/g | %ID/TO | Time (days) | No. animals | Average wt. (g) | %ID/g | %ID/TO | %ID/g | %ID/TO |
| 1 | 6 | 70 | 29·04 | 86·60 | 5·65 | 1·76 | 1 | 14 | 69 | 26·84 | 74·67 | 13·54* | 3·42* |
| | | | ±1·08 | ±2·87 | ±0·51 | ±0·40 | | | | ±1·48 | ±4·75 | ±1·94 | ±0·25 |
| 6 | 7 | 114 | 13·94 | 66·93 | 3·04 | 0·83 | 6 | 10 | 117 | 20·25* | 97·79* | 2·27 | 1·51 |
| | | | ±0·97 | ±2·19 | ±0·33 | ±0·12 | | | | ±0·91 | ±0·26 | ±0·33 | ±0·15 |
| 14 | 6 | 160 | 10·47 | 72·49 | 4·58 | 2·84 | 14 | 38 | 146 | 13·06 | 92·93* | 3·72 | 3·75 |
| | | | ±0·50 | ±2·60 | ±1·11 | ±0·96 | | | | ±1·04 | ±1·78 | ±0·33 | ±0·54 |

[a] Each recipient rat received a 0·2 ml saline suspension of $2 \times 10^4$ viable tumour cells intramuscularly or saline alone at 1, 6, and 14 days before evaluation.

[b] Hepatic and splenic phagocytic uptake of the colloid was determined at 10 min post-injection on both a per g (%ID/g) and total organ (%ID/TO) basis. The injected dose of $^{131}I$ test emulsion was 50 mg/100 g body weight. Mean ± s.e. mean are presented.

* Significant at the $P < 0{\cdot}05$ level or compared with timed control group.

FIG. 3.—Reticuloendothelial activity as the corrected phagocytic index (α) following tumour transplantation. Data are expressed as % control (α) with 6–7 controls evaluated at each time interval in a total of 70 rats. Similar to the experimental rats in Fig. 2, each point (●) represents an average of 7–18 tumour bearing rats with a total of 182 tumour rats evaluated. Phagocytic activity at 6, 14 and 30 days is significantly ($P < 0 \cdot 05$) different from controls.

FIG. 4.—Relative liver and spleen weight alterations in the tumour bearing rats injected intramuscularly with $2 \times 10^4$ viable tumour cells. Liver and spleen weights are expressed as % gross body weight (%GBW) and % net body weight (%NBW). NBW was calculated by subtracting the tumour weight from the GBW. The increased spleen size was significant ($P < 0 \cdot 05$) at the 14-day interval, while the increased liver size was significant ($P < 0 \cdot 05$) over the 14–30 day period. Control group consisted of 70 rats evaluated at various time intervals (6–7 per time), and tumour bearing group consisted of 7–18 animals at each point with a total group of 182.

The liver and spleen macrophage phagocytic uptake of the test colloid is presented in the Table at times of maximum RE alteration. Liver phagocytosis on both a per g and total organ basis manifested a slightly lower, but not significant, decrease by 24 h post-transplantation. Controls manifested a progressive decrease in colloid uptake per g liver as normally associated with liver growth. In contrast, the tumour bearing rats at 6 days and 14 days manifested increased ($P<0{\cdot}05$) hepatic Küpffer cell uptake. While not presented in the Table each period of increased hepatic uptake was associated with a decline in the pulmonary localization of the blood-borne test microparticles. Splenic uptake manifested a variable response with an unexpected intense hyperphagocytosis by 24 h. As seen from Fig. 3 and the Table, the alterations in RE clearance activity were primarily a reflection of the level of liver phagocytic capacity.

Presented in Fig. 5 is the circulating opsonic activity, assessed over the 30-day period. There was an excellent correlation between the opsonic activity (Fig. 5) and the observed state of the macrophage system (Fig. 3). Specifically, at 6 and 14 days there was a 93% and 99% elevation in the opsonin level respectively, in association with these 2 periods of RE stimulation. Moreover, the progressive decline in phagocytosis over the 14–30 day period (Fig.2) was associated with a significant ($P<0{\cdot}05$) fall in the opsonin activity, especially at 24 and 30 days when the activity was 45% and 52% of control levels which were $5{\cdot}80 \pm 0{\cdot}32$ %ID/100 mg. The apparent early decline in opsonic activity at 24 h was significant ($P<0{\cdot}05$) and may be related to the mild drop in phagocytic level detected at this time (Fig. 2, 3).

FIG. 5.—Plasma opsonic activity during tumour growth compared with saline injected controls. The control level represents the mean of all 89 determinations done over the 30-day period. These were pooled since a relatively constant level was obtained over the entire experimental period. All data are expressed as the mean ± standard error of the mean with the number of determinations in parentheses. Alterations at 1, 6, 14, 24 and 30 days were significant ($P<0{\cdot}05$). Opsonic activity of plasma is expressed in terms of its phagocytosis stimulatory capacity for *in vitro* Küpffer cell colloid phagocytosis during a 30-min incubation. Data expressed as % injected dose 2000 μg colloid dose (%ID) phagocytozed per 100 mg tissue.

### DISCUSSION

The reticuloendothelial system (RES) is endowed with the physiological capacity to rapidly phagocytoze foreign particulate matter, denatured endogenous proteins, tumour cells and effete autologous tissue debris (Biozzi and Stiffel, 1965; Saba, 1970*b*, 1975). The major portion of the RES consists of sessile macrophages localized in the liver, spleen and bone marrow which are in direct contact with the circulation. In this regard, the hepatic Küpffer cells functionally comprise approximately 80–90% of the total RE cell phagocytic clearance activity.

Evaluation of RE function in humans (Donovan, 1967; Salky *et al.*, 1974) has shown that a marked alteration in phagocytic activity occurs in patients with diseases of altered immunity, bacterial infections and neoplasia. These findings have accented the potential role of the RES as an antibacterial and anti-tumour defence mechanism. Indeed, RE stimulation will afford protection against experimentally induced infections (Biozzi and Stiffel, 1965; Saba, 1970*b*), as well as lead to a regression of tumour growth (Biozzi *et al.*, 1958; Diller *et al.*, 1963; Old *et al.*, 1959), while RE depression will decrease host resistance to infection and neoplastic disease (Biozzi *et al.*, 1958; Saba, 1970*b*).

Phagocytosis is intimately associated with the macrophage " recognition " of foreignness. Thus, macrophages can discriminate between foreign matter (non-self), altered endogenous tissue (altered-self), and healthy indigenous tissue (self) (Saba, 1970*b*). This capacity has been suggested to be, in part, related to plasma or serum factors (Allen *et al.*, 1973; Pisano *et al.*, 1972; Saba, 1975) which interact with the foreign or altered surface and stimulate phagocytosis. The delicate humoral control of the RES is emphasized by the fact that enhanced macrophage activity can be correlated with elevated opsonic or recognition factor levels, while depressed phagocytosis can be induced by lowering opsonic activity (DiLuzio *et al.*, 1972; Saba, 1972; Saba and DiLuzio, 1969). This *in vivo* sensitivity to the circulating opsonin level is especially manifested by the Küpffer cells of the liver (Saba, 1970*b*).

Macrophage defence mechanisms are involved in the host's response to counteract the growth and spread of cancer (Biozzi *et al.*, 1958; DiLuzio *et al.*, 1974*a*; Old *et al.*, 1960; Omori, 1964). Macrophage stimulation either before or during the early stages of tumour growth will inhibit tumour growth and spread; and depression of the macrophage system will accentuate tumour growth. For example, Halpern, Biozzi and Stiffel (1963) observed a clear protective influence of BCG infection against Sarcoma J in mice. They noted that the apparent increased resistance revealed by different strains of mice to tumour growth was correlated with activation of the macrophage system, suggesting that macrophage activation endows the host with increased capacity to destroy tumour cells. Additionally, activation of the macrophage system has been shown to be an important factor in the *C. parvum* inhibition of tumour growth (Wolmark and Fisher, 1974; Woodruff, Dunbar and Ghaffar, 1973) especially since selective inhibition of T and B cell function (Castro, 1973; Woodruff *et al.*, 1973) will not minimize the effectiveness of macrophage activation by *C. parvum* on tumour growth.

These findings do not prove a direct relationship between RES function and tumour growth but do suggest that the RES may exert a regulatory influence over the course and pattern of tumour development and growth. The findings by Stern (1960) on RES activity in inbred mice which manifest clear differences in the spontaneous incidence of malignant tumours further emphasizes the potential role of the RES in neoplasia. Thus, phagocytosis by hepatic Küpffer cells and splenic macrophages was greatest in animals manifesting the lowest incidence of spontaneous tumours, while animals exhibiting the greatest incidence of spontaneous tumours exhibited lower basal levels of RE activity. He postulated that macrophage "failure or weakness" may be a critical factor in tumour growth.

The present investigation has demonstrated that sequential phasic alterations of RES phagocytic capacity could be closely correlated with the circulating plasma level of recognition factor protein or opsonic protein. This was most pronounced at 6 and 14 days post-transplantation when the intense increment of plasma opsonin activity correlated with a a state of Küpffer cell hyperphagocytosis. Pisano *et al.* (1972) have recently demonstrated that patients with advanced carcinoma manifest very low opsonic or recognition factor levels. These human studies correlate well with the present animal findings, since the tumour bearing rats by 24–30 days post-transplantation, at a time of maximal tumour size and extensive metastases, manifest severe hypo-opsonaemia. Whether this is due to a depletion of plasma opsonic protein by the continual overload of the macrophage cells in terms of tumour cell clearance from the circulation, or possibly related to the sequestration of opsonic protein from blood to the site of tumour growth and associated tissue necrosis can only be speculated. The fact that intravenous injection of tumour cells will lead to a rapid decrease of the plasma opsonin activity (DiLuzio *et al.*, 1972; Saba *et al.*, 1974) as well as the observation that purified $^{125}I$ opsonic protein is sequestered from the vascular compartment into a site of tissue injury (Kaplan and Saba, 1974) supports either or both of these mechanisms. However, one must consider the possibility that the tumour may exert a depressive effect on the RES by inhibition of opsonic activity or impairment of cellular phagocytic capacity. Indeed, the recovery of opsonic protein activity in patients with metastatic disease following surgical removal of the tumour may indicate that its synthesis and/or activity is suppressed during tumour growth (Pisano, DiLuzio and Salky, 1970).

Old *et al.* (1960) demonstrated a striking relationship between reticuloendothelial function as reflected by carbon clearance and the growth of various transplanted and spontaneous tumours in mice. Thus, with the transplanted sarcoma 180 tumour in mice, there was minimal RES alteration within 4 days, followed by RES stimulation in association with splenic enlargement over the 7–12 day period. Thereafter, progressive diminution in phagocytic activity was associated with progressive tumour growth and decreased liver and spleen size. A similar response exists with the transplantable adenosarcoma 755 and associated with simultaneous enlargement of both the liver and spleen (Old *et al.*, 1960). Association of the hepatic and splenic enlargement with the enhanced RES clearance capacity was further suggested by studies utilizing the S180 ascites tumour model and the Friend virus leukaemic model (Old *et al.*, 1960). In the present findings, the hepatomegaly and splenomegaly were also observed and indeed correlated with the second peak of RES stimulation at 14 days. However, as presented in Fig. 2, 3 and 4, one cannot readily explain the profound alterations in clearance capacity throughout the experimental period on the basis of organ size alterations alone since no increment in relative liver and spleen weight was observed at the 4–6 day period during maximal RE stimulation. Moreover, the RES was hypophagocytic at the 30-day terminal stage, at a time of normal spleen size and enlarged liver size. In contrast, the parameter of opsonin or HRF levels (Fig. 5) correlated to a high degree throughout the experimental period with the functional state of the reticuloendothelial system (Fig. 2. 3). Thus, hyperopsonaemia was associated with enhanced colloid clearance capacity especially at 6 and 14 days, while hypo-opsonaemia was associated with the progressive period of RE failure. The previously reported inverse relationship between these 2 variables when adult rats are the tumour recipients (Kampschmidt and Pulliam, 1972) may reflect the known variation in RES response to tumour growth as influenced by age (Kampschmidt and Clabaugh, 1964).

In contrast to the opsonin level alterations in the tumour bearing rats, the phagocytic capabilities of the Küpffer cell remain relatively constant if tested *in vitro* in the presence of normal plasma (Saba, 1975; Saba *et al.*, 1974). Thus hepatic Küpffer cells obtained from normal rats as well as from animals at 6, 14, and 30 days after transplantation manifest similar phagocytic activity when incubated in normal plasma. In contrast, macrophages derived from both normal animals and from animals at various stages following tumour cell transplantation exhibit hyperphagocytosis when incubated in plasma from tumour bearing rats at 6 and 14 days and hypophagocytosis when incubated in plasma derived from tumour bearing rats at 30 days post transplantation. Thus, opsonic protein or so-called humoral recognition factor (HRF) appears to exert a regulatory role on the RES both in normal and in tumour bearing animals. A similar observation of humoral control on the RES has been made repeatedly especially with respect to RE alterations following surgery, whole body trauma, starvation and colloid-induced RE blockade (Saba, 1975, 1972; Saba and DiLuzio, 1969). Moreover, the passive administration of opsonic protein (Allen *et al.*, 1973) or the opsonization of particles before injection reverses the RE depression after blockade, surgery and starvation (Saba, 1970*a*; Saba and DiLuzio, 1969).

While the function of the RES correlates with the opsonin levels, it is much more difficult to explain the basis for the variations in opsonic activity during tumour growth. The abrupt increase in the opsonin level over the 4–6 day period may represent a host-defence response in which opsonic protein maintained in a storage pool is released into the vascular compartment (Saba, 1970*b*). The second peak of elevated opsonin levels may reflect increased synthesis of this factor in response to excessive consumption. In contrast, the progressive decline in the opsonin level during the terminal phase may reflect continual depletion of this factor from the plasma compartment in association with the vascular entrance of tumour cells (Saba and Antikatzides, 1972; Saba *et al.*, 1974).

The evidence supporting a role for the macrophage system in tumour immunity coupled with the intricate control that this alpha-2-globulin opsonic protein has on RE function provides the critical link necessary for the potential utilization of opsonic system manipulation as an approach for tumour therapy. This specific protein has been isolated and partially characterized (Allen *et al.*, 1973; Saba, 1975), and its passive administration may be of distinct benefit either separately or in conjunction with other modalities of cancer chemotherapy. The recent demonstration of inhibition of tumour growth by opsonic protein or HRF in animals (DiLuzio *et al.*, 1974) coupled with the regression of neoplastic lesions in humans by a combination of HRF and glucan administration supports this concept (DiLuzio *et al.*, 1974*a*). Indeed, these findings suggest that the alpha-2-globulin opsonic system (Saba, 1975) may be critical to the anti-tumour defence capacity of the macrophage system.

This study was supported by USPHS CA-16011 and ACS-IN-9M. We wish to thank Ms Maureen Kaiser for her assistance in the preparation of this manuscript.

### REFERENCES

ALLEN, C., SABA, T. M. & MOLNAR, J. (1973) Isolation, Purification and Characterization of Opsonic Protein. *J. reticuloendothel. Soc.*, **13**, 410.

BIOZZI, G. & STIFFEL, C. (1965) The Physiology of the Reticuloendothelial Cells of the Liver and Spleen. In *Progress in Liver Diseases.* Ed. H. Popper and F. Schaffner. New York: Grune and Stratton.

BIOZZI, B., STIFFEL, C., HALPERN, B. N. & MARTON. D. (1958) Étude de la fonction phagocytaire du S.R.E. aucours du developpment de tumeurs experimentales chez le rat et la souris. *Ann. Inst. Pasteur*, **94**, 681.

CASTRO, J. E. (1973) In *Ciba Foundation Symposium on Immunopotentiation.* Amsterdam: Associated Publishers.

DILLER, I. C., MANKOWSKI, Z. T. & FISHER, M. E. (1963) The Effect of Yeast Polysaccharides on Mouse Tumors. *Cancer Res.*, **23**, 201.

DILUZIO, N. R., MANSELL, P. W. A., MCNAMEE, R., KREMENTZ, E. T., ICHINOSE, H. & REED, R. J. (1974*a*) Macrophage Induced Necroses of Human Malignant Cells *in vivo*. *J. reticuloendothel. Soc.*, **16**, 37a.

DILUZIO, N. R., MCNAMEE, R., OLCAY, I., KITAHAMA, A. & MILLER, R. H. (1974*b*) Inhibition of Tumor Growth by Recognition Factors. *Proc. Soc. exp. Biol. Med.*, **145**, 311.

DILUZIO, N. R., MILLER, E., MCNAMEE, R. & PISANO, J. C. (1972) Alterations in Plasma Recognition Factor Activity in Experimental Leukemia. *J. reticuloendothel. Soc.*, **11**, 186.

DONOVAN, A. J. (1967) Reticuloendothelial Function in Patients with Cancer. *Am. J. Surg.*, **114**, 230.

HALPERN, B. N. BIOZZI, G. & STIFFEL, C. (1963) Action de L'extract microbien Wxb 3148 sur L'evolution des Tumerus Experimentates. In *Role du Systeme Reticuloendothelial dans L'Immun-*

*ité' Anti Bacterienne et Antitumoral.* Paris: Editions du Centre National de la Recherche Scientifique.

KAMPSCHMIDT, R. F. & CLABAUGH, (1964) Effect of Jensen Sarcoma upon the Reticuloendothelial System of Rats of Different Ages. *Proc. Soc. exp. Biol. Med*, **115**, 681.

KAMPSCHMIDT, R. F. & PULLIAM, L. A. (1972) Changes in the Opsonin and Cellular Influences on Phagocytosis during the Growth of Transplantable Tumors. *J. reticuloendothel. Soc.*, **11**, 1.

KAPLAN, J. E. & SABA, T. M. (1974) Localization of $^{125}$I-opsonic Protein in Injured Tissue following Surgery: Its Significance to Host Resistance to Circulating Malignant Cells. *J. reticuloendothel. Soc.*, **15**, 68a.

OLD, L. J., BENACERRAF, B., CLARKE, D. A., CARSWELL, E. A. & STOCKERT, E. (1961) The Role of the Reticuloendothelial System in the Host Reaction to Neoplasia. *Cancer Res.*, **21**, 1281.

OLD, L. J., CLARKE, D. A. & BENACERRAF, B. (1959) The Effect of Bacillus Calmette-Guerin Infection on Transplantable Tumours in the Mouse. *Nature, Lond.*, **184**, 291.

OLD, L. J., CLARKE, D. A., BENACERRAF, B. & GOLDSMITH, M. (1960) The Reticuloendothelial System and the Neoplastic Process. *Ann. N.Y. Acad. Sci.*, **88**, 264.

OMORI, Y. (1964) The Relation between the Reticuloendothelial System and the Spread of Cancer, especially in Gastric Carcinoma. *Tohoku J. exp. Med.*, **81**, 315.

PISANO, J. C., DILUZIO, N. R. & SALKY, N. K. (1970) Absence of Macrophage Humoral Recognition Factor(s) in Patients with Carcinoma. *J. Lab. clin. Med.*, **76**, 141.

PISANO, J. C., JACKSON, J. P., DILUZIO, N. R. & ICHINOSE, H. (1972) Dimensions of Humoral Recognition Factor Depletion in Carcinomatous Patients. *Cancer Res.*, **32**, 11.

SABA, T. M. (1970*a*) Mechanisms Mediating Reticuloendothelial System Depression after Surgery. *Proc. Soc. exp. Biol. Med.*, **133**, 1132.

SABA, T. M. (1970*b*) Physiology and Physiopathology of the Reticuloendothelial System. *Archs intern. Med.*, **126**, 1031.

SABA, T. M. (1972) Effect of Surgical Trauma on the Clearance and Localization of Blood-borne Particulate Matter. *Surgery, St. Louis*, **71**, 675.

SABA, T. M. (1975) Aspecific Opsonins. Proc. 4th Internat. Conv. on Immunology. In *Immune System and Infectious Diseases.* Basel: S. Karger Co. pp. 489.

SABA, T. M. & ANTIKATZIDES, T. G. (1972) Relationship of Macrophage Activity to Tumor Growth. *J. reticuloendothel. Soc.*, **11**, 396.

SABA, T. M. & DILUZIO, N. R. (1965) Küpffer Cell Phagocytosis and Metabolism of a Variety of Particles as a Function of Opsonization. *J. reticuloendothel. Soc.*, **2**, 437.

SABA, T. M. & DILUZIO, N. R. (1969) Reticuloendothelial Blockade and Recovery as a Function of Opsonic Activity. *Am. J. Physiol.*, **216**, 197.

SABA, T. M., ANTIKATZIDES, T. G. & LORENZEN, J. R. (1974) Phagocytosis Promoting Capacity of Plasma during Tumor Growth and Following the Vascular Entrance of Tumor Cells. *J. reticuloendothel. Soc.*, **15**, 18a.

SABA, T. M., FILKINS, J. P. & DILUZIO, N. R. (1966) Properties of the "Opsonic System" Regulating *in vitro* Hepatic Phagocytosis. *J. reticuloendothel. Soc.*, **3**, 398.

SALKY, N. K., DILUZIO, N. R., P'POOL, D. B. & SUTHERLAND, A. J. (1964) Evaluation of Reticuloendothelial Function in Man. *J. Am. med. Ass.*, **187**, 744.

SNELL, G. D. (1953) A Cytosieve Permitting Sterile Preparation of Tumor Cells for Transplantation. *J. natn. Cancer Inst.*, **13**, 1511.

STERN, K. (1960) The Reticuloendothelial System and Neoplasia. In *Reticuloendothelial Structure and Function.* New York: Ronald Press Co.

WOLMARK, N. & FISHER, B. (1974) The Effect of Single and Repeated Administration of *Corynebacterium parvum* on Bone Marrow Macrophage Colony Production in Syngeneic Tumor-bearing Mice. *Cancer Res.*, **34**, 2869.

WOODRUFF, M. F. A., DUNBAR, N. & GHAFFAR, A. (1973) The Growth of Tumours in T-cell Deprived Mice and Their Response to Treatment with *Corynebacterium parvum. Proc. R. Soc. Lond.*, **184**, 97.